# A Systematic Review of the Literature on the Current Revascularization Strategies for Aberrant Left Vertebral Artery During Total Endovascular and Hybrid Treatment of Aortic Arch Disease

**DOI:** 10.3390/jcm14217626

**Published:** 2025-10-27

**Authors:** Marta Minucci, Ottavia Borghese, Antonio Luparelli, Domenico Pascucci, Laura Rascio, Giovanni Tinelli, Tommaso Donati, Yamume Tshomba

**Affiliations:** 1 Unit of Vascular Surgery, Fondazione Policlinico Universitario A. Gemelli IRCCS, 00168 Rome, Italy; 2 Unit of Vascular Surgery, Department of Cardiovascular Sciences, Università Cattolica del Sacro Cuore, 20123 Rome, Italy; 3 Health Management, Fondazione Policlinico Universitario A. Gemelli IRCCS, 00168 Rome, Italy; 4 Department of Life Science and Public Health, Università Cattolica del Sacro Cuore, 20123 Rome, Italy

**Keywords:** aberrant vertebral arteries, aortic arch disease, TEVAR, complications, stroke, mortality

## Abstract

**Background/Objectives:** The aim of this study was to assess the current management strategies of Isolated Left Vertebral Artery (ILVA) arising directly from the aortic arch during total endovascular or hybrid repair of aortic arch pathologies and their safety and efficacy. **Methods:** A systematic literature review was undertaken to assess the current management strategies for ILVA during total endovascular or hybrid repair of aortic arch pathologies on three databases (PubMed, SCOPUS and Web of Science) from inception to February 2025, according to PICO and PRISMA guidelines (PROSPERO CRD42024562104). The safety (overall and aortic-related mortality; neurological complications) and efficacy (revascularization patency, endoleak and reintervention rate) of both approaches were investigated. **Results:** Out of 224 articles found, seven retrospective cohort studies (178 patients) were included. Overall, 149 patients (74.2% male, mean age 63 years) underwent ILVA revascularization. Two studies reported open ILVA revascularization through transposition; three studies reported endovascular revascularization strategies, and one study reported both open and endovascular techniques. The overall mortality rate was 1.3% at 30 days and 5.4% at a mean follow-up of 46 months (range 6–120) with a reported rate of aortic mortality of 0.7%. In the transposition group (55 patients), the rate of minor neurological complications was 16.6%, and the rate of major neurological complications was 7.3%; loss of patency rate was 16.3% and reintervention rate was 11.7%. Endovascularly treated patients (94 patients) experienced a rate of minor neurological complications of 2.1% and major neurological complications of 1%; the loss of patency rate was 2.1%, and the reintervention rate was 3.1%. **Conclusions:** Both surgical and endovascular techniques for ILVA revascularization seem to assure an acceptable rate of mortality and neurological complications during treatment of arch pathologies. However, currently available data are poor, non-standardized and based on single-center experiences. Therefore, until more robust data are available to indicate the superiority of one approach over another, the management strategies for aberrant ILVA should be individualized based on the anatomic characteristics and the center experience. Our findings underscore the need for prospective studies with standardized protocols.

## 1. Introduction

Aberrant origins of the supra-aortic trunk (SAT) are uncommon, with a prevalence of 18% in the general population, but a higher proportion (35%) in patients with thoracic aortic disease [1,2,3].

An Isolated Left Vertebral Artery (ILVA) arising directly from the aortic arch between the left common carotid artery (LCCA) and the left subclavian artery (LSA) is described to be the second most frequent variant of SAT configurations, with an incidence ranging between 0.8% and 6.3% [1,2,3,4] and a left-side predominance [4].

Despite anatomical variations not being clinically relevant, their recognition is pivotal during treatment of cerebrovascular or thoracic aortic pathology, as their non-recognition and accidental ligation or lesion may lead to possibly devastating neurologic complications [5,6].

Proper understanding of anatomical variation is especially mandatory when planning endovascular and hybrid interventions involving the aortic arch, as those variants may represent significant technical challenges and deeply impact surgical strategy [7]. Several approaches have been described for ILVA management during endovascular and hybrid aortic arch repair, including transposition to the left carotid artery or total endovascular revascularization with dedicated fenestration [8,9,10].

The safety and efficacy of these approaches remain unproven due to the lack of large studies, long-term results, and follow-up data.

In this scenario, we investigated the current management strategies for aberrant left vertebral artery during total endovascular and hybrid repair of aortic arch pathologies with the aim of evaluating the effectiveness of such approaches, making the available evidence more accessible to decision makers in real-world clinical practice.

## 2. Methods

### 2.1. Study Design

This systematic review was conducted according to the guidelines of the Preferred Reporting Items for Systematic Reviews and Meta-Analyses (PRISMA) [11] and PICO (Patients, Intervention, Comparator, Outcomes) [12] approach (Supplementary Materials Tables S1 and S2; Figure S1).

A literature search was performed by two independent authors (MM and OB) based on three databases (PubMed, SCOPUS and WEB OF SCIENCE) from inception to February 2025 using the following string: (“isolated left vertebral artery” OR “ILVA” OR “aberrant left vertebral artery”) AND (“management” OR “treatment” OR “reconstruction” OR “transposition” OR “hybrid” OR “endovascular” OR “fenestration” OR “repair” OR “reparation” OR “TEVAR” OR “reimplantation” OR “replacement” OR “revascularization” OR “procedure”) AND (“safety” OR “follow-up” OR benefits OR effect OR effects OR efficacy OR outcome OR outcomes OR “process assessment” OR appropriateness OR “quality”).

All papers reporting the management (conservative or interventional) of ILVA during hybrid or endovascular treatment of aortic arch diseases (including aneurysm, chronic/acute dissection, intramural hematomas or penetrating aortic ulcer) were included as detailed below.

The titles and abstracts were screened for appropriateness and then a second round of eligibility was applied to the retrieved full text. Disagreement was resolved by discussion and consensus, and the reasons for exclusion were documented.

The reference lists of all included studies were also examined for identification of further relevant articles.

The International Prospective Register of Systematic Reviews (PROSPERO) was inquired to avoid duplication and the full protocol of the study was registered (CRD42024562104).

### 2.2. Endpoints

Primary and secondary outcomes were defined according to the SVS reporting standards for endovascular aortic repair of aneurysms involving the renal-mesenteric arteries [13].

Our primary endpoint was to investigate the different interventional strategies (endovascular or open) of aberrant ILVA arising directly from the aortic arch during endovascular and hybrid treatment of aortic arch diseases and the related mortality (overall and aortic-related at 30 days and during follow-up).

As secondary outcomes, we investigated new onset (minor and major) neurological symptoms (see definition above), immediate and follow-up patency rate of the ILVA revascularization, the occurrence of endoleak and ILVA-related reintervention rate.

### 2.3. Definitions

New onset neurological symptoms were divided into minor and major neurological symptoms. Minor neurological symptoms were defined as transient isolated brainstem symptoms such as balance disorders, dizziness, diplopia, dysphagia, aphasia, drop attacks and bilateral homonymous hemianopia.

Major neurological symptoms were defined as permanent neurological deficit, including anterior and posterior circulation stroke, paraparesis and paraplegia.

### 2.4. Inclusion and Exclusion Criteria

Papers were included if:

- An isolated left aberrant vertebral artery was detected on a contrasted computed tomography angiography (CTA) preoperatively;
- The left aberrant ILVA arose directly from the aortic arch;
- The included patients were treated for an aortic arch pathology (including aneurysm, chronic/acute dissection, intramural hematomas or penetrating aortic ulcer) with an endovascular or hybrid treatment either in an elective or emergent setting.

Only retrospective or prospective series, in English language and reporting sufficient data about management strategies and related outcomes, were included.

Literature review studies, conference abstracts, theses, case reports, case series, dissertations and book chapters were not included in our analysis.

### 2.5. Data Collection

The following data were retrieved from the included papers in a predefined database: authors, year of publication, country, study design, overall number of patients, number of patients presenting with ILVA, patients’ demographics, type of aortic arch disease, type of treatment, type of endograft used, setting for repair, 30-day and long-term mortality (for all causes and aortic-related), new neurological symptoms, aortic-related reinterventions patency of the revascularization, and the follow-up length.

In patients undergoing surgical or endovascular revascularization of the ILVA, the immediate and long-term patency rate and overall endoleak rate were also reported. No further search was conducted to retrieve any unpublished data.

### 2.6. Assessment of Study Quality and Risk of Bias

The studies were analyzed in terms of design, heterogeneity, and possible bias. As there were no randomized studies, papers reporting insufficient data or at high risk of bias according to the Newcastle Ottawa-Score (NOS) [14] were ruled out from the analysis (Supplementary Materials Table S3).

The selected articles were tested with the ROBINS-E risk of bias tool for non-randomized trials (Supplementary Materials Table S4).

### 2.7. Statistical Analysis

Categorical data are presented as counts and percentages. Continuous variables are reported as mean and range. Taking into consideration the small number of patients, no subgroups were analyzed, and no further statistical analyses were performed. Because of the heterogeneity of reported data, no meta-analysis of pooled data was performed. The extracted data are reported in percentages and absolute values or narratively synthesized. SPSS statistical software (v. 25, SPSS Inc., Chicago, IL, USA) was used for all analyses.

## 3. Results

### 3.1. Study Selection and Characteristics

**Figure 1:**
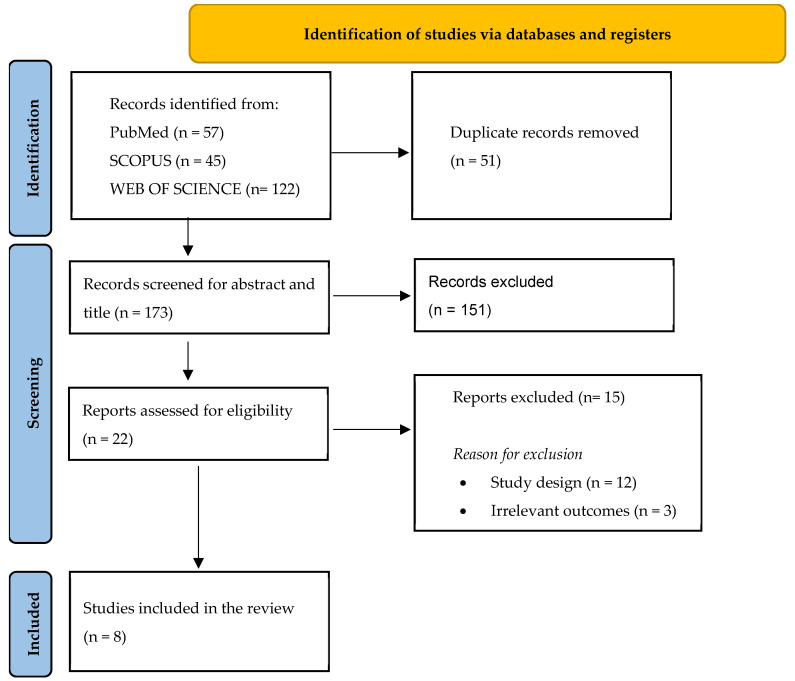
PRISMA flowchart. From: Page MJ, McKenzie JE, Bossuyt PM, Boutron I, Hoffmann TC, Mulrow CD, et al. The PRISMA 2020 statement: an updated guideline for reporting systematic reviews. BMJ 2021;372:n71 [11].

The search yielded 224 articles, and after duplication removal, a total of 173 papers were screened for title and abstract (Figure 1). Twenty-two papers were retrieved and assessed for text eligibility, among which eight (two retrospective multicenter cohort studies and six retrospective single-center cohort studies) were included according to the above-mentioned inclusion and exclusion criteria, totaling 199 patients.

The main characteristics of the included papers are reported in Table 1.

**Figure 2:**
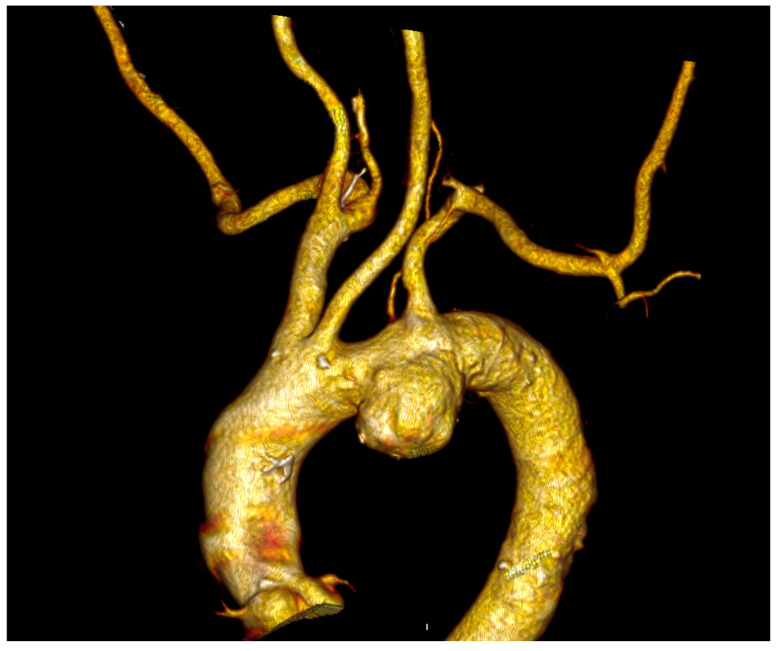
Preoperative Computed Tomography Angiography of a patient with a left vertebral artery arising directly from the aortic arch and affected with an aortic arch aneurysm treated at our institution.

In all cases, the ILVA was detected on the preoperative computed tomography angiography (CTA) (Figure 2).

Additionally, in 145 (73%) cases, a preoperative CTA of the brain was performed to rule out other concomitant diseases or anatomical variants and check the integrity of the Willis circle.

The classification of the vertebral artery variable origin defined by Lazaridis and colleagues [4] was used in only two studies [15,17] that reported LA2.2 configuration in all cases (19 patients).

Only six included studies reported the aortic stent-graft (SG) used during TEVAR for a total of 33 Castor single-branched stent grafts (MicroPort Medical, Shanghai, China), 21 TAG (W.L. Gore & Associates, Flagstaff, AZ, USA), 23 Valiant (Medtronic, Minneapolis, MN, USA), 14 Ankura (Lifetech Scientific, Shenzhen, China), 6 Zenith (Cook, Bloomington, IN, USA). 8 Aegis (MicroPort, Shanghai, China) and 2 RelayPro (Terumo Aortic, Tokyo, Japan).

### 3.2. Indication for Revascularization

Among the 199 patients with ILVA who underwent total endovascular or hybrid treatment of aortic arch disease, 149 patients (75% male; mean age 63 years, range 55–76 years) required additional procedures for ILVA revascularization (Table 2).

The underlying aortic arch disease was as follows: acute or chronic thoracic aortic dissection (80 patients, 53.7%), thoracic aortic aneurysm (28 patients, 18.9%), intramural hematoma (10 patients, 6.7%), aortic pseudoaneurysm (5 patients, 3.3%), penetrating aortic ulcer (5 patients, 3.3%) or not disclosed (21 patients, 14%).

The indication for revascularization was given for TEVAR requiring coverage of ILVA origin and one of the following reasons:

- dominant ILVA (54 cases, 36.2%) [9,10,17];
- symmetric vertebral arteries with an incomplete circle of Willis (45 cases, 30.2%) [9,10,17];
- increased risk of spinal cord ischemia for extensive coverage of the aorta (>200 mm or previous aortic surgery) (2 cases, 1.3%) [17].

A systematic transposition of the ILVA was performed in the remaining cases (48 cases, 32.2%) [8,15,18].

Conversely, in 50 cases ILVA was not revascularized due to:

- hypoplastic LVA (vertebral artery diameter < 2.0 mm) or right vertebral artery dominance (17 patients, 34%) [9];
- ILVA not involved in the aortic pathology or not covered by the stent-graft when Ishimaru zone 3-TEVAR was performed (32 patients, 64%) [15,16] or for chronic occlusion at the origin (1 patient, 2%) [15].

These patients were excluded from the analysis.

### 3.3. Strategies for Revascularization

**Figure 3:**
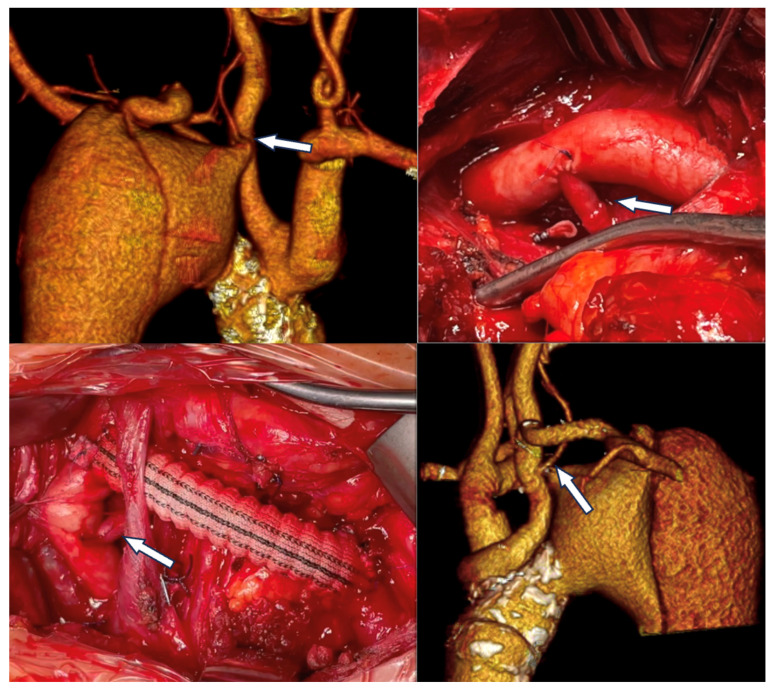
Left vertebral to left carotid transposition and left carotid-subclavian bypass to achieve adequate proximal landing zone for endovascular treatment of a chronic post-dissection aneurysm of the thoracic aorta in a patient with a left vertebral artery arising directly from the aortic arch (arrow) in a patient treated at our institution.

Several strategies for ILVA revascularization have been described in the included studies: the studies by Piffaretti et al., Yang et al., and Shergill et al. described the results achieved with ILVA open surgical revascularization during TEVAR (40 patients) [8,15]; the studies by Shen et al., Luo et al., and Wang et al. focused on total endovascular reconstruction through physician-modified fenestration (PMF) (39 patients) and in situ fenestration (ISF) (3 patients) [10,17,18]. Finally, Zhang and colleagues compared the outcomes of fenestration (24 patients), transposition (15 patients) and chimney technique (28 patients) for ILVA revascularization (Figure 3) [9].

#### 3.3.1. Open Surgical Revascularization

Overall, a total of 55/149 (37%) patients underwent open surgical revascularization of the ILVA as depicted following:

In case of zone 2 TEVAR:

- A single-stage intervention with revascularization of the left subclavian artery through a carotid-subclavian bypass and transposition of the ILVA onto the bypass graft or directly onto the left common carotid artery was performed in 27% of cases (40/149 patients).
- ILVA revascularization through a saphenous vein bypass from the left carotid was performed in 5.3% of cases (8/149 patients) through a supraclavicular approach.
- In 3 cases (2%), the method of vertebral revascularization was unclear.

In patients who underwent open arch graft replacement (4/149, 2.7%),

- Two (1.3%) underwent subsequent ILVA transposition;
- Two (1.3%) were treated with the so-called ‘‘arch first’’ graft technique in which ILVA transposition was performed during TEVAR, 2 to 4 weeks following open surgery [17].

#### 3.3.2. Endovascular Revascularization

**Figure 4:**
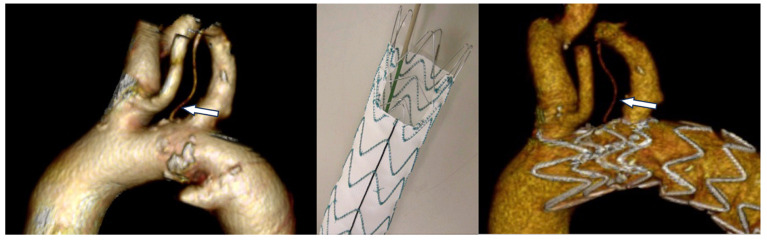
Endovascular treatment of a type Stanford B aortic dissection using a zone II scalloped TEVAR in a patient treated at our institution and presenting with an isolated left vertebral artery (arrow) revascularized through the scallop designed to preserve flow in the left subclavian artery.

Endovascular revascularization was performed in a total of 94/149 (63%) patients:

- physician-modified fenestration (PMF) was used in 63 patients (42.3%);
- chimney technique was performed in 28 patients (19%);
- in situ fenestration (ISF) was selected in 3 patients (2%) (Figure 4).

Among patients who underwent PMF, 33 had zone 2 TEVAR and ILVA revascularization via on-table fenestration of a Castor (MicroPort Medical, Shanghai, China) single-branch stent graft [10,18]; 30 of them underwent TEVAR with single or double on-table fenestrations for ILVA and subclavian artery revascularization (with the use of a bridging bare metal stent in only three cases) [9,17].

The 28 patients treated with chimney technique underwent a novel approach based on the delivery of a chimney stent in the LSA, ensuring that it crosses the LVA origin to guarantee its perfusion through the mesh of the stent and the gutter between the LSA chimney and the aortic stent-graft [9].

Finally, three cases of ISF were performed using a liver biopsy needle through a surgical supraclavicular approach for exposure of ILVA with a bare metal bridging stent [17].

### 3.4. Primary Endpoints

The overall 30-day mortality was 1.3% (one patient in the surgical revascularization group died during hospital admission for a ruptured thoracic aneurysm, and one patient in the endovascular group died 29 days after the procedure from a hemodialysis-related brain hemorrhage).

Among the remaining 147 patients, the late mortality rate was 5.4% (mean follow-up of 46 months, range 6–120 months), as two additional deaths occurred in the endovascular group (2/93, 2.1%) and six in the surgical group, respectively (6/54, 11.1%). Causes of death were respiratory failure in oxygen-dependent chronic respiratory insufficiency (one), advanced liver cancer (one), or not disclosed (six). No late aortic-related deaths were reported (Table 3).

### 3.5. Secondary Endpoints

#### 3.5.1. New Onset Neurological Symptoms

New onset neurological symptoms occurred in 11.4% of patients (17/149) as detailed following.

Minor neurological symptoms occurred in 7.4% of patients (16.4% in the open revascularization group and 2.1% in the endovascular group).

Major neurological symptoms were reported in 3.3% of patients (7.4% in the open revascularization group and 1% in the endovascular group). Specifically, one patient experienced an anterior stroke and another a posterior stroke (both patients had undergone transposition); one stroke localization was not disclosed (and occurred in a patient who had undergone PMF); one patient suffered from paraparesis and another from paraplegia (both patients had undergone transposition).

Furthermore, Horner syndrome was observed in one patient who had undergone surgical ILVA transposition; in this case, the central or peripheral etiology of the symptom was not disclosed [1].

#### 3.5.2. Secondary ILVA-Related Reintervention

Secondary ILVA-related interventions were needed in 5 patients symptomatic for dizziness who presented with ILVA stenosis during the follow-up period [9] (the reintervention rate was 11.7% in the surgical group and 3.1% in the endovascular group, respectively).

Among them, a stenting procedure was performed in two patients initially treated with IFS, while angioplasty was performed in three patients who had previously undergone transposition. These data were calculated excluding the paper from Shergill et al. Due to the inability to disambiguate reintervention data from this publication.

No further reinterventions were conversely performed in the subgroup of patients experiencing ILVA revascularization occlusion during follow-up (11/149 cases, 7.3%) (16.3% in the surgical group and 2.1% in the endovascular group respectively), of which nine were asymptomatic cases and two presented with dizziness. 

#### 3.5.3. Endoleak

Eleven cases of endoleaks (EL) were observed (11.7%) in the endovascular group. Notably, 5/28 (17.8%) patients treated with chimney technique experienced a type IA EL that was closely followed up without need for any additional intervention, and 6/63 (9.5%) having undergone PMF presented a type IA EL (four) or IIIC EL (two), resulting in one reintervention for bridging stent positioning.

Reported data in the included studies were insufficient to disambiguate the relationship between the underlying aortic pathology, or the anamnestic characteristics, and the outcomes achieved with the different vascular interventions performed.

## 4. Discussion

In recent years, endovascular strategies in association or not with rerouting of the supra-aortic vessels have been increasingly applied to treat arch and descending thoracic aorta pathologies. Indeed, these approaches mitigate the peri- and postoperative morbidity and mortality rates that have lately been reported to be about 5.7% for open surgery versus 1.9% for endovascular treatment, respectively [7].

Hence, the understanding of the anatomic variant of aortic arch vessels has become even more important to allow proper preoperative planning for the endovascular procedure.

ILVA has been described to be the second most frequent variant of SAT configurations [1,2,3,4]. With the steady increase in the number of endovascular procedures being performed worldwide, it is expected that vascular surgeons will face more and more such anatomical variants. However, whether ILVA should be revascularized or not in all cases is unclear. Several authors suggest a systematic revascularization of the ILVA in all cases [8,15,16]. When neurological risk for the brain or spinal cord is increased the revascularization of an aberrant ILVA is always indicated [8,15,16,20,21]. Indeed, other authors advocate for revascularization of the ILVA only under specific circumstances. This is the case of dominant ILVA during zone 2 TEVAR,9,10,15 incomplete circle of Willis or when an extensive coverage of the descending thoracic aorta is planned with occlusion of the intercostal vessels [17].

The current management strategies for revascularization of ILVA during endovascular/hybrid treatment of aortic arch diseases include both open and endovascular options, but no sufficient data from comparative studies are currently available to indicate the definitive superiority of one revascularization technique over another. According to the data presented above, an anatomical revascularization of the ILVA (both via open reimplantation or fenestrated technology) was the technique more frequently applied, as it guarantees a straightforward flow to the target vessels. Chimney technique and extra-anatomical surgical bypasses seem indeed to be burdened with potentially higher risk of patency loss during follow-up [22].

Endovascular approaches represented the preferred option in most of the included centers (63% of treated cases), probably because patients selected for these strategies since the beginning are poor candidates for any kind of open surgery or because a total endovascular intervention allows for ILVA revascularization during the index procedure avoiding the risk associated with surgical dissection (i.e., vagus/recurrent laryngeal nerve palsy, Horner’s syndrome and lymphocele) [23,24,25,26].

However, the frequency of reporting should not be conflated with superiority over open strategies. Indeed, despite reported data being too heterogeneous to draw any definitive conclusion, no aortic-related death was reported in any cases regardless of the intervention performed.

A recent retrospective study by Shergill and colleagues compared the results achieved in 143 patients who underwent vertebral artery revascularization during TEVAR, dividing patients into a direct vertebral revascularization cohort and an indirect (carotid-subclavian bypass or through subclavian-carotid transposition) vertebral revascularization cohort. The authors reported a significantly higher incidence of complications in the direct group than in the indirect one (bypass thrombosis 33.3% vs. 0.8%, *p* < 0.0001; and hoarseness 57.1% vs. 18.0%, *p* < 0.001, respectively), but there was no significant difference in mortality rates at 30 days, 1, 3, 5, and 10 years of follow-up [19].

Long-term data are yet to be defined for both techniques, but it seems that even in the case of occlusion of the reconstruction during follow-up, patients mostly remain asymptomatic, and no further intervention is required. Considering the frequent asymptomatic nature of vertebral revascularization occlusion and the rate of neurological complications, albeit predominantly minor, associated with both surgical and endovascular procedures, a more selective strategy for ILVA revascularization may be warranted.

Data currently available do not allow us to analyze nor compare the results achieved with these two techniques in the long term nor to completely comment on the comparative neurological outcomes.

As a result of what emerged from the analysis of the literature, we believe that until further and more robust data are available, when it is indicated to perform the revascularization of an ILVA, the procedure should be tailored to the anatomic characteristics of the patient, their comorbidity, and the setting of the procedure but also based on the center experience.

## 5. Conclusions

Overall, our findings underscore the need for prospective studies with standardized protocols. However, despite data currently available being extremely heterogeneous to draw any definitive conclusion, it seems that both surgical and endovascular techniques for ILVA revascularization during total endovascular and hybrid treatment of aortic arch pathologies allow for an acceptable rate of mortality and neurological complications.

Until more robust data are available to indicate the superiority of one strategy over another, the revascularization technique for ILVA should be tailored based on the anatomic characteristics of the patient and the center experience.

## 6. Limitation

This study is inherently limited by the non-standardized nature of the cases selected for analysis, which affects the uniformity of variables of interest. To start with, the findings are primarily based on single-center experiences, which may introduce procedural bias related to specific expertise or preferred techniques at those centers. The rarity of the condition also limits the available sample size, reducing the overall strength of the study. Additionally, a direct comparison between the two groups is not feasible, further weakening the robustness of the conclusions.

Moreover, most of the included studies were of fair methodological quality, with common limitations such as incomplete data, lack of control groups, and underreporting of complications. Indeed, some articles imprecisely reported or did not clearly specify neurological outcomes, making interpretation difficult.

Altogether, these factors limit the generalizability of our findings and highlight the need for prospective studies employing standardized protocols. Therefore, the reported results should be interpreted with caution.

## Figures and Tables

**Table 1:** Characteristics of included studies.

First Author	Country	Study Design	Total Sample Size (*n*)	Number of Revascularized ILVA	Age (Median and Range)	Median Follow-up (Months)	ILVA Management	Mortality Rate (Early + Late)	Neurological Complications (*n*/%)	Endoleak (*n*%)	ILVA-Related Reintervention (*n*%)	Patency Rate * (%)
Piffaretti et al., 2019 [15]	Italy	Cohort	8	6	75 y (range 56–83)	15	6 Transposition	25%	1 (16.7)	0	0 (0)	100
Yang et al., 2021 [8]	China	Cohort	13	13	61 y (range 42–71)	22	13 Transposition	0%	0 (0)	0	0 (0)	100
Ding et al., 2019 [16]	China	Cohort	31	0	55 y (range 31–66)	33	Conservative	3%	0 (0)	1 (3.2)	NA	NA
Shen et al., 2023 [17]	China	Cohort	9	9	60 y (range 38–76)	38	3 PMF 3 ISF	11%	0 (0)	0	0 (0)	78
Zhang et al., 2022 [9]	China	Cohort	84	67	60 y (range 37–77)	64	3 PMF 28 Chimney 15 Transposition	0%	7 (10.4)	9 (10.7)	5 (6)	97
Luo et al., 2024 [10]	China	Cohort	25	25	62.5 y (range 51–68)	28.5	25 PMF	4%	1 (4)	2 (8)	0 (0)	100
Wang et al., 2023 [18]	China	Cohort	8	8	62.5 y (range 41–75)	48	8 PMF	0%	0 (0)	0	0 (0)	100
Shergill et al., 2024 [19]		Cohort	21	21	73 y (range 58–75)	120	10 Transposition 8 Bypass 3 Surgical (not specified)	28.6%	8 (38)	NA	NA	66.7

* Patency rate at follow-up among the revascularized ILVAs. ILVA: isolated left vertebral artery; PMF: physician-modified fenestration; ISF: in-situ fenestration.

**Table 2:** Demographic, anatomical, clinical and operative details of patients with revascularized ILVA.

Demographics	N Patients 149 (%)
**Sex**	
Male	112 (75.2)
Female	37 (24.8)
**Age**	64 years (range 55–76)
CLINICAL DATA	
Indication for TEVAR *	
Thoracic aortic aneurysm	28 (21.8)
Thoracic aortic dissection (chronic/acute)	80 (62.5)
IMH	10 (7.8)
Aortic pseudoaneurysm	5 (3.9)
PAU	5 (3.9)
**Management strategy for ILVA**	N patient 199 (%)
Conservative management	50 (25.1)
Interventional management	149 (74.9)
Indication for ILVA revascularization	N patients 149 (%)
Dominant	54 (36.3)
Incomplete Willis	45 (30.2)
Extensive coverage of the aorta	2 (1.3)
Systematic	48 (32.2)
OPERATIVE DETAILS	
**ILVA’s revascularization technique**	
**Surgical**	55 (37)
Transposition	44 (29.7)
Extra-anatomical bypass	8 (5.3)
Not disclosed	3 (2)
**Endovascular**	94 (63)
PMF	63 (42)
ISF	3 (2)
Chimney	28 (19)
Setting for repair **	
Emergent/Urgent	22 (23.4)
Elective	72 (76.6)
**Type of stent-graft used ^‡^**	
Castor single-branched stent graft (MicroPort Medical, China)	33 (30.8)
TAG (W.L. Gore & Associates, Flagstaff, AZ, USA)	21 (19.7)
Valiant (Medtronic, Minneapolis, MN, USA)	23 (21.5)
Ankura (Lifetech Scientific, Shenzhen, China)	14 (13)
Zenith (Cook, Bloomington, IN, USA)	6 (5.6)
Aegis (MicroPort, Shanghai, China) RelayPro (Terumo Aortic)	8 (7.4) 2 (1.8)

Data are reported as n (%) or mean/range. * Data obtained from 7 studies (n = 128 patients), due to the lack of data about indication for treatment in 1 study. ** Data obtained from 3 studies (n = 94 patients), due to the lack of data about setting for repair in 4 studies. ^‡^ Data obtained from 6 studies (n = 107 patients) due to the lack of data concerning the type of thoracic stent-graft used in 2 studies and the partial data reported from another study. TEVAR: thoracic endovascular aortic aneurysm repair; IMH: Intramural Haematoma; PAU: Penetrating Aortic Ulcer; ILVA: isolated left vertebral artery; LVA: left vertebral artery; PMF physician modified fenestration; ISF: in-situ fenestration.

**Table 3:** Technical and clinical outcomes of ILVA revascularized patients during the in-hospital period and follow-up.

Clinical Outcomes	N Patients 149 (%)
**Aortic related mortality**	1 (0.7)
**Overall mortality**	
**Early (within 30 days from the index procedure)**	2 (1.3)
Open revascularization group	1/55 (1.8)
Endovascular group	1/94 (1)
**Late (>30 days after the index procedure)**	8(5.4)
Open revascularization group	6/54 (11.1)
Endovascular group	2/93 (2.1)
**New onset neurological symptoms**	11.4(17)
**Minor neurological symptoms**	12 (8)
Open revascularization group	9/54 (16.6)
Endovascular group	2/94 (2.1)
**Major neurological symptoms**	5 (3.4)
Open revascularization group	4 (7.4)
Endovascular group	1/94 (1)
**Follow-up data** (mean follow-up of 46 months, range 6–120)	
ILVA occlusion	11(7.3)
ILVA stenosis	5 (3.3)
Endoleak	11(11.7)
ILVA-related reintervention	5 (3.3)
Open revascularization group	11/94 (11.7)
Endovascular group	3/94 (3.1)

Data are reported as n (%) or mean/range. Percentage calculated on available data from survivors after 30 days. Mean follow-up of 46 months (range 6–120 months) months. Including type Ia and IIIc endoleak.

## Data Availability

No new data were created or analysed in this study.

## Supplementary Materials

The following supporting information can be downloaded at: https://www.mdpi.com/article/10.3390/jcm14217626/s1, Table S1: PRISMA checklist (Moher et al. 2009). Table S2: PICO framework. Table S3: Risk of Bias Assessment. Newcastle-Ottawa Scale Items. Table S4: Risk Of Bias in Non-randomized Studies – of Exposures (ROBINS-E) assessment.

## Abbreviations

The following abbreviations are used in this manuscript.

CTA	computed tomography angiography
ILVA	isolated left vertebral artery
IMH	intramural haematoma
ISF	in-situ fenestration
LCCA	left common carotid artery
LSA	left subclavian artery
PAU	penetrating aortic ulcer
PMF	Physician-modified fenestration
SAT	supra-aortic trunk
TEVAR	thoracic endovascular aortic aneurysm repair
