# Supplementary material for: A Systematic Review of the Literature on the Current Revascularization Strategies for Aberrant Left Vertebral Artery During Total Endovascular and Hybrid Treatment of Aortic Arch Disease
Source: J Clin Med. 2025 Oct 27;14(21):7626. doi: 10.3390/jcm14217626 (PMC12608799; doi:10.3390/jcm14217626)
Supplement: Supplementary file 1 [file jcm-14-07626-s001.zip › jcm-3849877-supplementary.pdf]

Supplementary table S1. PRISMA checklist (Moher et al. 2009)

Section/topic	#	Checklist item	Reported on page #
TITLE			
Title	1	Identify the report as a systematic review, meta-analysis, or both.	1
ABSTRACT			
Structured Summary	2	Provide a structured summary including, as applicable: background; objectives; data sources; study eligibility criteria, participants, and interventions; study appraisal and synthesis methods; results; limitations; conclusions and implications of key findings; systematic review registration number.	2
INTRODUCTION			
Rationale	3	Describe the rationale for the review in the context of what is already known.	3
Objectives	4	Provide an explicit statement of questions being addressed with reference to participants, interventions, comparisons, outcomes, and study design (PICOS).	3
METHODS			
Protocol and registration	5	Indicate if a review protocol exists, if and where it can be accessed (e.g., Web address), and, if available, provide registration information including registration number.	4
Eligibility criteria	6	Specify study characteristics (e.g., PICOS, length of follow-up) and report characteristics (e.g., years considered, language, publication status) used as criteria for eligibility, giving rationale.	4-5
Information sources	7	Describe all information sources (e.g., databases with dates of coverage, contact with study authors to identify additional studies) in the search and date last searched.	3-4
Search	8	Present full electronic search strategy for at least one database, including any limits used, such that it could be repeated.	3-4

Study selection	9	State the process for selecting studies (i.e., screening, eligibility, included in systematic review, and, if applicable, included in the meta-analysis).	3-4
Data collection process	1 0	Describe method of data extraction from reports (e.g., piloted forms, independently, in duplicate) and any processes for obtaining and confirming data from investigators.	5
Data items	1 1	List and define all variables for which data were sought (e.g., PICOS, funding sources) and any assumptions and simplifications made.	3
Risk of bias in individual studies	1 2	Describe methods used for assessing risk of bias of individual studies (including specification of whether this was done at the study or outcome level), and how this information is to be used in any data synthesis.	5
Summary measures	1 3	State the principal summary measures (e.g., risk ratio, difference in means).	5
Synthesis of results	1 4	Describe the methods of handling data and combining results of studies, if done, including measures of consistency (e.g., I2) for each meta-analysis.	5
Risk of bias across studies	1 5	Specify any assessment of risk of bias that may affect the cumulative evidence (e.g., publication bias, selective reporting within studies).	N/A
Additional analyses	1 6	Describe methods of additional analyses (e.g., sensitivity or subgroup analyses, meta-regression), if done, indicating which were pre-specified.	N/A
RESULTS			
Study selection	1 7	Give numbers of studies screened, assessed for eligibility, and included in the review, with reasons for exclusions at each stage, ideally with a flow diagram	6
Study characteristic s	1 8	For each study, present characteristics for which data were extracted (e.g., study size, PICOS, follow-up period) and provide the citations.	6
Risk of bias within studies	1 9	Present data on risk of bias of each study and, if available, any outcome level assessment (see item 12).	6
Results of individual studies	2 0	For all outcomes considered (benefits or harms), present, for each study: (a) simple summary data for each intervention group (b) effect estimates and confidence intervals, ideally with a forest plot.	N/A

Synthesis of results	21	Present results of each meta-analysis done, including confidence intervals and measures of consistency.	N/A
Risk of bias across studies	22	Present results of any assessment of risk of bias across studies (see Item 15).	N/A
Additional analysis	23	Give results of additional analyses, if done (e.g., sensitivity or subgroup analyses, meta-regression [see Item 16]).	N/A
DISCUSSION			
Summary of evidence	24	Summarize the main findings including the strength of evidence for each main outcome; consider their relevance to key groups (e.g., healthcare providers, users, and policy makers).	10
Limitations	25	Discuss limitations at study and outcome level (e.g., risk of bias), and at review-level (e.g., incomplete retrieval of identified research, reporting bias).	16
Conclusions	26	Provide a general interpretation of the results in the context of other evidence, and implications for future research.	14-15
FUNDING		This research received no specific grant from any funding agency in the public, commercial, or not-for-profit sectors.	16
Funding	27	Describe sources of funding for the systematic review and other support (e.g., supply of data); role of funders for the systematic review.	N/A

Supplementary table S2. PICO framework

PICO ELEMENTS	Keywords	Search terms
**P (Patient or Population)**	Patients with aberrant left vertebral artery and aortic arch disease	Isolated left vertebral artery, ILVA
**I (Intervention)**	Endovascular revascularization of aberrant left vertebral artery	Management, treatment, hybrid, endovascular, fenestration, repair, reparation, TEVAR, procedure
**C (Comparison)**	Surgical revascularization of aberrant left vertebral artery	Transposition, reconstruction, reimplantation, replacement, revascularization
**O (Outcomes)**	Aortic-related mortality, complication, new neurological symptoms, stroke rate, reintervention rate	Safety, outcomes, follow-up, benefits, effects

Supplementary table S3. Risk of Bias Assessment. Newcastle-Ottawa Scale Items

First author, Year	Representativeness of the exposed cohort	Selection of the non-exposed cohort	Ascertainment of exposure	Demonstration that outcome of interest was not present at start of study	Comparability of cohorts on the basis of the design or analysis	Assessment of outcome	Was follow-up long enough for outcomes to occur	Adequacy of follow up of cohorts	Total score
Piffaretti et al, 2019	1	1	1	1	0	1	1	1	7
Yang et al, 2021	1	0	1	1	0	1	1	1	6
Ding et al 2019	1	0	1	1	0	1	1	1	6
Shen et al 2023	1	0	1	1	0	1	1	1	6
Zhang et al 2022	1	1	1	1	1	1	1	1	8
Luo et al 2024	1	0	1	1	0	1	1	1	6
Wang et al 2023	1	0	1	1	0	1	1	1	6

Shergill et al, 2024	1	0	1	1	1	1	1	1	7

None of the retrospective studies were considered as low quality with a high risk of bias according to the Newcastle–Ottawa Scale assessment.

Supplementary table S4. Risk Of Bias in Non-randomized Studies – of Exposures (ROBINS-E) assessment.

First author, Year	**D1. Confounding**	**D2. Selection**	**D3. Exposure classification**	**D4. Deviations**	**D5. Missing data**	**D6. Outcome measurement**	**D7. Report selection**	**Overall judgement**
Piffaretti et al, 2019	Critical – case series, no comparator, very small sample	Moderate – single center, selected patients	Low – surgical transposition well described	Moderate – standardized but varied	Low – follow-up reported (median 15 months)	Low – objective, patency and neurological events	Moderate – focus on feasibility and safety	Serious
Yang et al, 2021	Serious – retrospective, small sample, no comparator	Moderate – selected cases, criteria based on guidelines	Low – hybrid TEVAR + ILVA transposition well described	Moderate – standardized but varied	Low – follow-up reported (median 22 months)	Low – objective, imaging and clinical outcomes	Moderate – focus on feasibility and safety	Serious
Ding et al 2019	Critical – no comparator, treatment algorithm introduces bias by indication	Moderate – rare ILVA cases from large TBAD cohort	Low – zone definitions and techniques clearly described	Moderate – co-interventions variable but guided by protocol	Low – follow-up reported (median 33 months)	Low – objective imaging and clinical outcomes	Moderate – focus on safety and reintervention	Serious
Shen et al 2023	Serious – retrospective,	Moderate – inclusion/exclusion criteria	Low – PMF and ISF technique	Moderate – protocol	Low – follow-up reported	Low – objective imaging and	Moderate – focus on	Serious

	small sample, wo techniques compared without adjustment	narrow, possible bias	s clearly defined	described, no crossover	(median 38 months)	clinical outcomes	patency and safety	
Zhang et al 2022	Moderate – multicenter but not randomized	Moderate – inclusion/exclusion criteria may bias, only certain anatomies included	Low – three operative strategies clearly described	Moderate – procedural variability across centers	Moderate – follow-up up to 64 months, unclear completeness	Low – objective imaging and neurological outcomes	Moderate – endpoints predefined but limited functional outcomes	Moderate
Luo et al 2024	Serious – multicenter series, no comparator, patients highly selected	Moderate – clear inclusion/exclusion, selected anatomies	Low – Castor + PMF technique clearly described	Moderate – standardized procedures, minor variations	Low – follow-up reported (mean 28.5 months)	Low – CTA-based objective outcomes	Moderate – focused on patency, safety, reintervention	Serious
Wang et al 2023	Critical – case series, no comparator, very small sample	Moderate – selected patients with ILVA and unfavourable anatomy	Low – Castor fenestration procedure clearly defined	Moderate – standardized but varied	Low – follow-up reported (up to 72 months)	Low – objective imaging and clinical outcomes	Moderate – focus on technical and safety	Serious
Shergill et al, 2024	Moderate – retrospective, comparative; adjustments reduced but did not eliminat	Moderate – single center, consecutive inclusion but referral bias possible	Low – direct vs indirect revascularization well defined	Moderate – surgeon discretion in technique choice, possible co-interve	Low – follow-up reported (up to 120 months)	Moderate – mortality and stroke objective, but some outcomes (hoarseness)	Moderate – main outcomes reported ,	Moderate

	e confounding			ntion differences		subjective		
